# miRNAs mediate SnRK1-dependent energy signaling in *Arabidopsis*

**DOI:** 10.3389/fpls.2013.00197

**Published:** 2013-06-20

**Authors:** Ana Confraria, Cláudia Martinho, Alexandre Elias, Ignacio Rubio-Somoza, Elena Baena-González

**Affiliations:** ^1^Plant Stress Signaling, Instituto Gulbenkian de CiênciaOeiras, Portugal; ^2^Department of Molecular Biology, Max Planck Institute for Developmental BiologyTübingen, Germany

**Keywords:** *Arabidopsis*, SnRK1, stress, miRNA, energy signaling, DCL1

## Abstract

The SnRK1 protein kinase, the plant ortholog of mammalian AMPK and yeast Snf1, is activated by the energy depletion caused by adverse environmental conditions. Upon activation, SnRK1 triggers extensive transcriptional changes to restore homeostasis and promote stress tolerance and survival partly through the inhibition of anabolism and the activation of catabolism. Despite the identification of a few bZIP transcription factors as downstream effectors, the mechanisms underlying gene regulation, and in particular gene repression by SnRK1, remain mostly unknown. microRNAs (miRNAs) are 20–24 nt RNAs that regulate gene expression post-transcriptionally by driving the cleavage and/or translation attenuation of complementary mRNA targets. In addition to their role in plant development, mounting evidence implicates miRNAs in the response to environmental stress. Given the involvement of miRNAs in stress responses and the fact that some of the SnRK1-regulated genes are miRNA targets, we postulated that miRNAs drive part of the transcriptional reprogramming triggered by SnRK1. By comparing the transcriptional response to energy deprivation between WT and *dcl1-9*, a mutant deficient in miRNA biogenesis, we identified 831 starvation genes misregulated in the *dcl1-9* mutant, out of which 155 are validated or predicted miRNA targets. Functional clustering analysis revealed that the main cellular processes potentially co-regulated by SnRK1 and miRNAs are translation and organelle function and uncover TCP transcription factors as one of the most highly enriched functional clusters. TCP repression during energy deprivation was impaired in miR319 knockdown (*MIM319*) plants, demonstrating the involvement of miR319 in the stress-dependent regulation of *TCP*s. Altogether, our data indicates that miRNAs are components of the SnRK1 signaling cascade contributing to the regulation of specific mRNA targets and possibly tuning down particular cellular processes during the stress response.

## Introduction

The ability of an organism to respond to challenges in the energy status is critical for its survival. Energy supplies vary over time and therefore sophisticated mechanisms have evolved to monitor fluctuations in nutrient availability and to manage adequately storage compounds, allowing the maintenance of an energy balance at the cellular and whole-organism levels. In plants, energy deficiency can be the result of impaired carbon assimilation and/or respiration upon exposure to adverse environmental conditions (Baena-Gonzalez and Sheen, 2008).

The decline in cellular ATP levels ensuing stress activates the evolutionarily conserved SnRK1 protein kinases, metabolic sensors closely related to the budding yeast Snf1 (sucrose-non-fermenting) and mammalian AMPK (AMP-activated Protein Kinase), and encoded by three genes in the *Arabidopsis* genome (*SnRK1.1/1.2/1.3*) (Hardie, 2007; Polge and Thomas, 2007). In response to energy deficiency SnRK1s trigger extensive metabolic and transcriptional changes that contribute to restoring homeostasis and to elaborating adequate longer-term acclimation strategies (Baena-Gonzalez et al., 2007). This includes activation of catabolic pathways such as autophagy and breakdown of starch, and the inhibition of anabolic pathways like translation. In addition to metabolic readjustment, SnRK1 coordinates stress-induced responses, and plants with diminished SnRK1 activity are severely impaired in their response to a wide array of stresses such as flooding, sudden darkness, salinity, and biotic stress (Hao et al., 2003; Lovas et al., 2003; Schwachtje et al., 2006; Baena-Gonzalez et al., 2007; Lee et al., 2009).

SnRK1s control metabolism partly through direct phosphorylation and inactivation of key enzymes such as sucrose phosphate synthase, nitrate reductase and HMG-CoA reductase (Halford et al., 2003). In addition, SnRK1s induce transcriptional changes in over a thousand genes implicated in energy metabolism, cell signaling, defence and stress (Baena-Gonzalez et al., 2007), but the mechanisms underlying this mode of regulation are still poorly understood. A few key bZIP transcription factors and common *cis*-elements have been identified downstream of SnRK1 (Baena-Gonzalez et al., 2007), regulating genes involved in primary metabolism such as *ASPARAGINE SYNTHETASE1* (*ASN1/DIN6*), *PROLINE DEHYDROGENASE* (*ProDH1* and *ProDH2*) and others, and hence inducing alterations in the corresponding metabolites (Hanson et al., 2008). Studies on the yeast Snf1 and the mammalian AMPK, on the other hand, have uncovered multiple modes of action beyond control of transcription factors, and these protein kinases have been shown to affect transcription through the direct association with chromatin and through the interaction with different components of the transcriptional machinery, including the SRB/mediator complex (Hedbacker and Carlson, 2008; McGee and Hargreaves, 2008).

microRNAs (miRNAs) are endogenous single-stranded non-coding RNAs of 20–24 nt length that act *in trans* on non-self RNAs, negatively regulating their expression post-transcriptionally (Bartel, 2009). Plant miRNAs act through cleavage of highly complementary mRNA targets, but also through translational repression and chromatin modification (Mallory and Bouche, 2008; Voinnet, 2009). In *Arabidopsis*, they are encoded by nuclear *MIR* genes, transcribed into primary miRNAs (*MIR* transcripts) that are processed by a nuclear-localized complex containing DICER-LIKE1 (DCL1), exported to the cytoplasm and incorporated into an ARGONAUTE (AGO) containing RNA-induced silencing complex (RISC) for recognition of mRNA targets with a complementary sequence (Voinnet, 2009). Molecular, genetic and biochemical analysis have demonstrated that miRNAs play central roles in growth, development, and morphogenesis (Jones-Rhoades et al., 2006; Axtell et al., 2007). In addition, genome-wide deep sequencing and microarray profiling have identified nutrient- (Hsieh et al., 2009; Pant et al., 2009; Liang et al., 2012; Ren and Tang, 2012) and stress-responsive miRNAs (Jones-Rhoades and Bartel, 2004; Sunkar and Zhu, 2004; Zhou et al., 2007, 2008; Hewezi et al., 2008; Liu et al., 2008; Moldovan et al., 2010; Licausi et al., 2011; Zhang et al., 2011), which have accordingly been suggested as important mediators of these adaptive processes (Ruiz-Ferrer and Voinnet, 2009; Khraiwesh et al., 2012; Sunkar et al., 2012). A global connection between miRNAs and stress has also been suggested from the fact that miRNA biogenesis mutants exhibit altered responses to multiple types of stress as well as hypersensitivity to ABA, a key regulator of stress responses (Lu and Fedoroff, 2000; Kim et al., 2008; Zhang et al., 2008; Li et al., 2012). The relevance of specific miRNAs for stress tolerance has also been demonstrated for several miRNAs, including miR398, miR393, or miR169 (Navarro et al., 2006; Sunkar et al., 2006; Li et al., 2008).

Furthermore, several miRNAs, including miR319, miR156, miR159, and miR172, seem to respond similarly to rather diverse types of environmental conditions, ranging from biotic stress to drought, hypoxia and UV light (Sunkar et al., 2012). In some situations a link to the cellular energy status has been established: (1) chemical inhibition of mitochondrial respiration induces a similar set of miRNAs as hypoxia stress in *Arabidopsis* roots (Moldovan et al., 2010); (2) oxidative stress and sucrose have opposite effects on the accumulation of miR398, also involved in copper homeostasis (Dugas and Bartel, 2008). Altogether, this prompted us to postulate that miRNAs could act as downstream effectors of the SnRK1 pathway contributing to the reprogramming of gene expression executed by SnRK1.

Here, we present a comparative microarray profiling of WT and *dcl1–9* plants under control and energy-deficiency conditions. We identify a set of 831 genes with compromised regulation in the *dcl1–9* mutant, a fraction of which (19%) are validated or predicted miRNA targets, including the miR319 targets *TCP2* and *TCP4*.

## Materials and methods

A list of all primers used in this study is provided in Table S1.

### Plant material and growth conditions

All *Arabidopsis thaliana* (*Arabidopsis*) plants used in this study are in the Columbia (*Col*-0) background. *dcl1–9* was originally isolated in ecotype *Wassilewskija* (Feldmann, 1992) and has been backcrossed five times to *Col*-0 ecotype (Vazquez et al., 2004). *dcl1–9* plants were propagated as heterozygotes and the *DCL1–9*/*dcl1–9* heterozygous plants were distinguished from *DCL1–9*/*DCL1–9* homozygous plants by PCR amplification of genomic DNA with specific T-DNA and *DCL1* primers (Table S1). Homozygous *dcl1–9/dcl1–9* plants were isolated from the segregating *DCL1–9/dcl1–9* progeny based on their phenotype (Jacobsen et al., 1999; Kidner and Martienssen, 2004). Plants overexpressing a miR319 target mimic (*MIM319*) were used as miR319 knockdown plants. These and the corresponding empty vector pGREEN control have been previously described (Franco-Zorrilla et al., 2007). Sterilized seeds were stratified in the dark at 4°C for 2 days, and sowed on plates containing 0.5× Murashige and Skoog medium with 0.1% MES, 0.8% phytoagar, and 1% sucrose. Plates were sealed and incubated under a photoperiod of 12 h light (100 μE; 22°C)/12 h dark (18°C). After 10 days, seedlings were transferred to 1:3 vermiculite:soil mixture and kept in the same photoperiod and temperature conditions.

### Energy stress treatment

For energy stress treatment 5 week-old plants were used. Under these conditions no bolting was observed for any of the genotypes. Well-expanded leaves (true leaf numbers 5–7) were detached and incubated in Petri dishes with 20 mL of sterile MilliQ water for 6 h either in the light or in the dark. When indicated, dark incubation was carried out in the presence of 50 mM glucose. Dark treatment started 2 h after the onset of the light period. After the treatment, leaves were quickly pat-dried, flash-frozen in liquid *N*_2_ and kept at −80°C until RNA extraction.

### Microarray analyses

All the microarray gene expression data reported in this study are available from the NCBI Gene Expression Omnibus (GEO) database (accession number GSE46713).

The transcriptomic response of WT and *dcl1–9* plants to 6 h of unexpected darkness was compared using microarrays. Total RNA from three biological replicates was extracted using TRIzol® reagent (Life Technologies) and treated with RNase-Free DNase (Promega). The concentration and purity of DNased RNA were determined by spectrophotometry and integrity was confirmed using an Agilent 2100 Bioanalyzer with a RNA 6000 Nano Assay (Agilent Technologies, Palo Alto, CA).

RNA was processed for use on Affymetrix (Santa Clara, CA, USA) *Arabidopsis* Gene 1.1 ST Array Strips by using the Ambion WT Expression Kit (Life Technologies, CA, USA) and Affymetrix GeneChip WT Terminal Labeling Kit, according to the manufacturer's protocols. Briefly, 100 ng of total RNA containing spiked in Poly-A RNA controls (GeneChip Expression GeneChip Eukaryotic Poly-A RNA Control Kit; Affymetrix) was used in a reverse transcription reaction (Ambion WT Expression Kit) to generate first-strand cDNA. After second-strand synthesis, double-stranded cDNA was used in an *in vitro* transcription (IVT) reaction to generate cRNA (Ambion WT Expression Kit). 15 μg of this cRNA was used for a second cycle of first-strand cDNA synthesis (Ambion WT Expression Kit). 5.5 μg of single stranded cDNA was fragmented and end-labeled (GeneChip WT Terminal Labeling Kit; Affymetrix). Size distribution of the fragmented and end-labeled cDNA, respectively, was assessed using an Agilent 2100 Bioanalyzer with a RNA 6000 Nano Assay.

3.5 μg of end-labeled, fragmented cDNA was used in a 150 μl hybridization cocktail containing added hybridization controls (GeneAtlas Hybridization, Wash, and Stain Kit for WT Array Strips, Affymetrix), of which 120 μl were hybridized on array strips for 20 h at 48°C. Standard post hybridization wash and double-stain protocols (GeneAtlas Hybridization, Wash, and Stain Kit for WT Array Strips, Affymetrix) were used on an Affymetrix GeneAtlas system, followed by scanning of the array strips.

### Data analysis

Scanned arrays were analyzed first with Affymetrix Expression Console software for quality control. Subsequent analysis was carried out with DNA-Chip Analyzer (dChip; http://www.dchip.org, Wong Lab, Harvard) using custom cdf file aragene11st_At_TAIRG.cdf and respective annotations as available from Brainarray database version 15 (Vazquez et al., 2004; Sandberg and Larsson, 2007). The arrays were normalized to a baseline array with median CEL intensity by applying an Invariant Set Normalization Method (Li and Wong, 2001). Normalized CEL intensities of the 12 arrays were used to obtain model-based gene expression indices based on a Perfect Match-only model (Li and Hung Wong, 2001). Replicate data for the same sample type were weighted gene-wise by using inverse squared standard error as weights. All genes compared were considered to be differentially expressed if the 90% lower confidence bound of the fold-change between experiment and baseline was above 1.2. The lower confidence bound criterion means that we can be 90% confident that the fold-change is a value between the lower confidence bound and a variable upper confidence bound. Li and Hung Wong (2001) have shown that the lower confidence bound is a conservative estimate of the fold-change and therefore more reliable as a ranking statistic for changes in gene expression.

### Comparison of microarray experiments and profile intersection

In order to validate the 6 h-dark incubation of WT and *dcl1–9* leaves as a *bona fide* starvation treatment, we crossed-compared our datasets with a previously published set of 600 core starvation genes (core SGs), obtained by a stringent comparison of microarray data from SnRK1.1 overexpression in protoplasts with that of several conditions that impact the cellular energy status [Table S4 in Baena-Gonzalez et al. (2007)]. Genes were considered as similarly regulated if they were up-regulated in the core SG set and also in the 6 h-dark treatment by at least 1.2-fold [lower confidence bound of the fold-change, (Li and Hung Wong, 2001)]. Similar criteria were applied to the downregulated genes.

For the global intersection of the WT and *dcl1–9* profiles with that of SnRK1 activation, the SnRK1 microarray dataset was filtered using the described criteria (Baena-Gonzalez et al., 2007) but a lower cut-off of 1.5-fold-change. Overlap between the compared datasets was revealed using the Venny Venn diagram on-line application (http://bioinfogp.cnb.csic.es/tools/venny/index.html; Oliveros, 2007).

### List of miRNA targets

A list of validated and predicted targets was used as compiled from literature and from public databases [ASRP and *Arabidopsis* MPSS and PARE databases (Nakano et al., 2006; Backman et al., 2008; German et al., 2008) by Folkes et al. (2012)]. In addition, psRNAtarget (Dai and Zhao, 2011), TargetFinder (Allen et al., 2005; Fahlgren et al., 2007), and the UEA Plant Target Prediction tools (Moxon et al., 2008) were used with the default parameters to predict further targets for all the *Arabidopsis* miRNA sequences deposited in the miRBase database (Kozomara and Griffiths-Jones, 2011). TAIR10 annotation was used as a reference.

### Quantitative real-time RT-PCR

DNase-treated RNA was reverse transcribed (1 μg) using SuperScript III Reverse Transcriptase (Life Technologies), following the manufacturers' instructions. qRT–PCR analyses were performed in a 7900HT Fast Real-Time PCR System (Applied Biosystems), using the iTaq™ Universal SYBR® Green Supermix (BioRad), and the 2^−ΔΔ*Ct*^ method for relative quantification (Livak and Schmittgen, 2001). Expression values were normalized using the CT values obtained for the *EIF4* (*At3g13920*) and *ACT2* (*At3g18780*) control genes, in experiments employing detached leaves or protoplasts, respectively.

### Transient expression assays in protoplasts

Protoplast transient expression assays were carried out as previously described, using freshly isolated cells from mature fully expanded leaves (Baena-Gonzalez et al., 2007; Yoo et al., 2007). The *35S::SnRK1.1-HA*, *proDIN6::LUC*, and *proDIN1(SEN1)::LUC* constructs have been described elsewhere (Baena-Gonzalez et al., 2007; Ramon et al., in press). The latter two employ firefly *LUCIFERASE* (*LUC*) fused to the indicated promoters as a reporter. For qRT-PCR analysis, 12 × 10^5^ protoplasts were transfected either with 1.2 mg of *35S::SnRK1.1-HA* or control DNA. For generating the *proMIR161::LUC* and *proMIR775::LUC* reporter constructs the genomic sequences upstream of the predicted fold-back structures of *MIR161* (−3086 bp) and *MIR775* (−1853 bp) were cloned using the primers listed in Table S1 and used to replace the *DIN6* promoter in the *proDIN6::LUC* reporter. For promoter activity assays, 2 × 10^4^ protoplasts were co-transfected with 10 μg reporter construct, 10 μg *35S::SnRK1.1-HA* effector construct/control DNA and 0.5 μg *UBQ10::GUS* as transfection control. After transfection, protoplasts were incubated for 6 h under light, harvested by centrifugation, flash-frozen in dry ice and kept at −20°C for LUC/GUS analyses or used immediately for RNA extraction. Luciferase and glucuronidase activities were measured as previously described (Yoo et al., 2007).

### Functional classification and annotation

Functional classification and annotation of relevant sets of genes was carried out using the Database for Annotation, Visualization and Integrated Discovery (DAVID) v6.7 (Huang Da et al., 2009), accessible online at http://david.abcc.ncifcrf.gov/

### Statistical analyses

All statistical analyses and the associated graphs were performed with the GraphPad Prism software. For analyses of qRT-PCR data, the statistical significance of the indicated changes was assessed employing log_2_ transformed relative expression values (Rieu and Powers, 2009).

## Results

### Global response in WT and *dcl1–9* plants to energy depletion and SnRK1 activation

The SnRK1 protein kinase was shown to be activated in response to energy depletion and conversely to be inactivated by sugar (Baena-Gonzalez et al., 2007). Upon activation, SnRK1 triggers extensive transcriptional changes. To test whether a lower energy status and a concomitant SnRK1 activation could trigger differential gene expression through miRNA action, we first established a starvation experiment in WT and *dcl1–9* mutant plants. To this end, we kept mature detached leaves of WT and *dcl1–9* in the dark for 6 h and performed microarray analyses employing the *Arabidopsis* 1.1 ST array strip (Table S2). Expression values were normalized, modeled and analyzed using dChip software. Genes were considered to be differentially expressed if the 90% lower confidence bound of the fold-change between experiment and baseline was above 1.2. The lower confidence bound is a conservative estimate of the fold-change and therefore more reliable as a ranking statistic for changes in gene expression (Li and Hung Wong, 2001; Li and Wong, 2001). We selected a 1.2 cut-off for two main reasons: (1) comparative microarray-based studies between WT and weak miRNA biogenesis mutants like *dcl1–9* have reported rather mild changes in the steady-state levels of numerous established miRNA targets (Ronemus et al., 2006; Laubinger et al., 2010), (2) transient gene expression changes attributed to miRNA action in response to external stimuli are often within the 20–40% range (Navarro et al., 2006; Moldovan et al., 2010).

**Figure 1:**
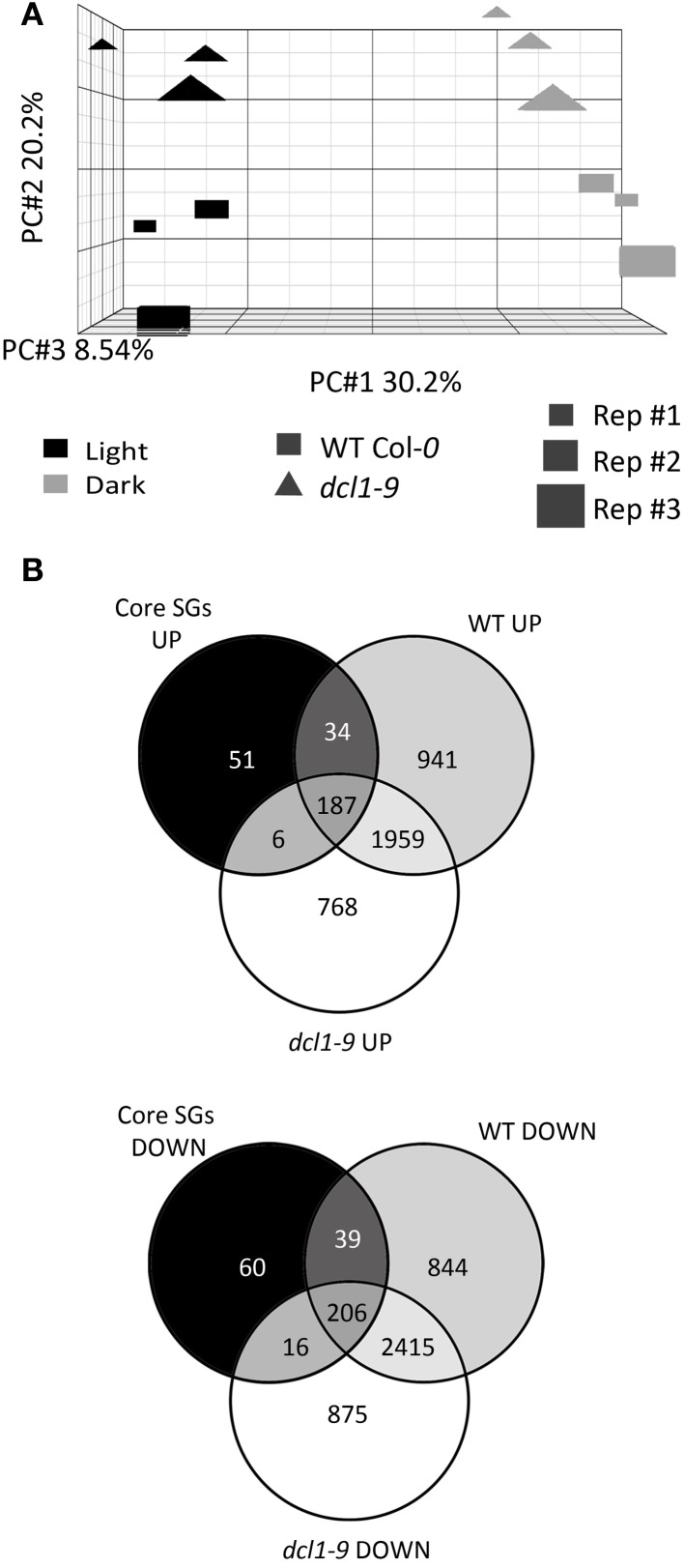
**The *dcl1–9* mutant is partially compromised in the overall transcriptional reprogramming induced by starvation. (A)** The dark-triggered transcriptional response encompasses more differences in WT than in *dcl1–9* plants. Principal component analysis of normalized microarray data was performed using PARTEK®. The *X* axis represents the distance between control and dark-treated samples, the *Y* axis represents the distance between WT and *dcl1–9* plants and the *Z* axis represents the distance between the 3 independent biological replicates. **(B)** Dark treatment triggers a “starvation response” in WT and *dcl1–9*, which is partially deficient in *dcl1–9*. Venn diagrams illustrate the intersection between genes activated (UP) or repressed (DOWN) similarly by SnRK1 activation and various starvation conditions [core starvation genes, core SGs, Table S4 in Baena-Gonzalez et al. (2007)], and genes significantly activated or repressed by darkness in WT and *dcl1–9* leaves.

Principal component analysis (PCA) of the microarray experiment revealed that the main variable generating differences across the datasets is the dark treatment (30.2% of the variation), and that the differences induced in the *dcl1–9* background are less pronounced than in the WT (Figure 1A). In order to validate the 6 h-dark exposure as a *bona fide* starvation treatment, we compared the transcriptional changes induced by exposure to sudden darkness in WT and *dcl1–9* plants with a set of “core starvation genes” (core SGs), previously identified as commonly regulated by the SnRK1 protein kinase and various treatments that affect the energy status [Table S4 in Baena-Gonzalez et al. (2007)] (Figure 1B). The dark treatment provoked to a large extent a “starvation response” in the WT, as evidenced by the fact that 466 of the 600 core SGs (78%) were at least 1.2-fold (lower bound of fold-change) similarly regulated in response to darkness. The dark treatment also induced a “starvation response” in the *dcl1–9* mutant, although the number of core SGs similarly regulated by darkness was lower than in the WT (415 genes, 69%). This comparison also shows that within the 466 core SGs similarly regulated by darkness in the WT, 73 (16%) were not responsive in *dcl1–9*. Altogether, these differences are in agreement with the PCA analysis, and suggest that compromised DCL1 function results in a partial deficiency in the starvation response and that miRNAs may be involved in the regulation of starvation genes.

### Energy depletion and SnRK1 activation reduce the levels of specific *MIR* transcripts

The *Arabidopsis* 1.1 ST array strip contains probes for 170 *MIR* genes and therefore, we examined their expression as a first step to investigate the possible contribution of miRNAs to the starvation response. Indeed, in addition to changes in the core SGs, exposure to darkness triggered significant changes in several *MIR* transcripts, causing a reduction e.g., in *MIR398C*, *MIR414*, and *MIR775A* or an accumulation, as in the case of *MIR172A*, *MIR419*, and *MIR404* (Table S3).

**Figure 2:**
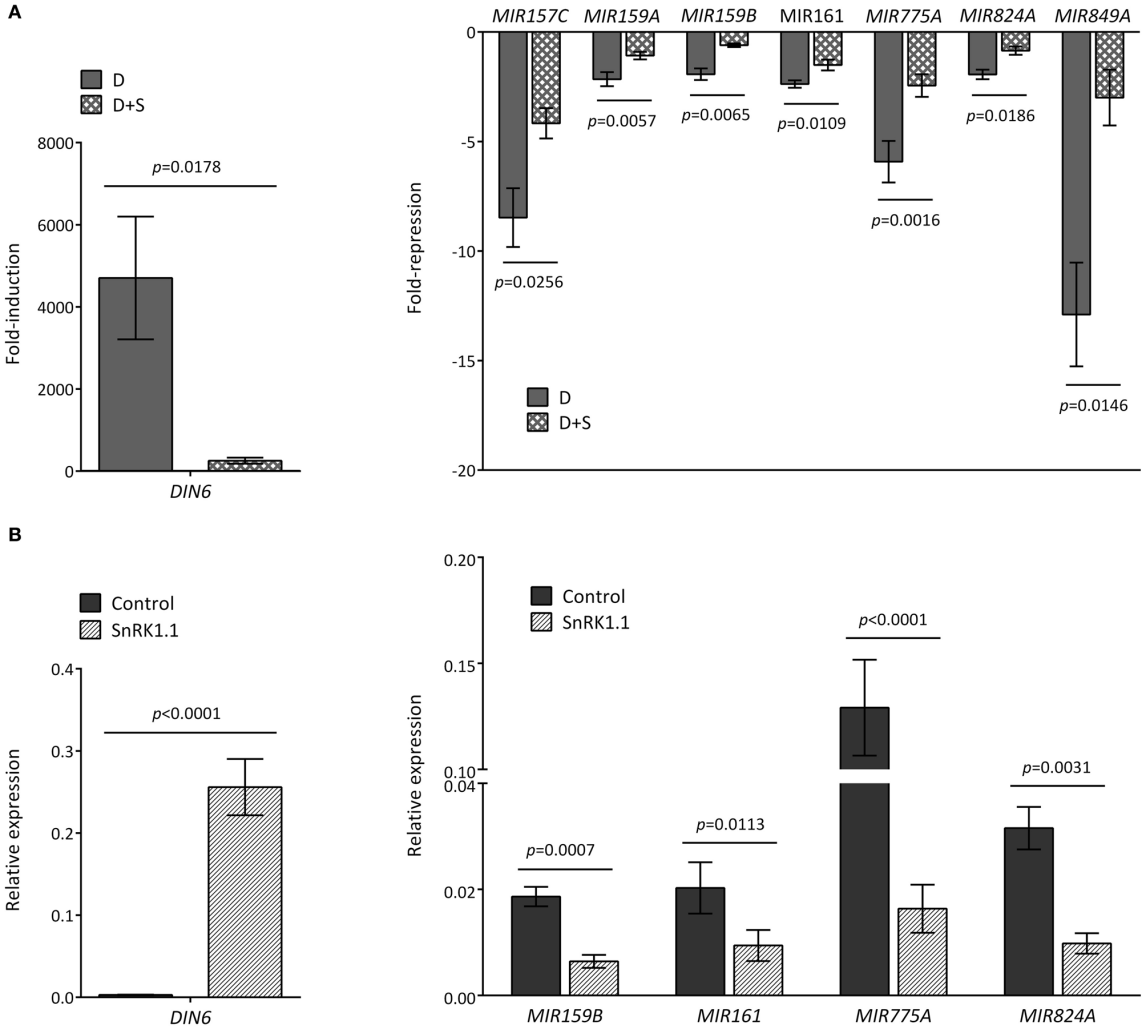
**Reduced accumulation of *MIR* transcripts in response to darkness and SnRK1 activation relies partly on the energy status. (A)** The energy status contributes to the decline of *MIR* transcripts in dark-treated leaves. Values represent fold-repression of *MIR* transcripts in the dark (D) and dark+sugar (D+S) relative to the light control. **(B)** SnRK1.1 activation in mesophyll protoplasts causes a reduction in *MIR* transcript levels. Values represent relative transcript levels upon transient overexpression of SnRK1.1 or control DNA. The induction of the SnRK1 marker gene *DIN6* serves as control of SnRK1 activation by darkness **(A)** and SnRK1.1 overexpression **(B)**. Relative mRNA levels were assessed by qRT-PCR, error bars represent the standard error of the mean (SEM) from at least three independent experiments. *p*-values, paired *t*-test.

**Figure A1:**
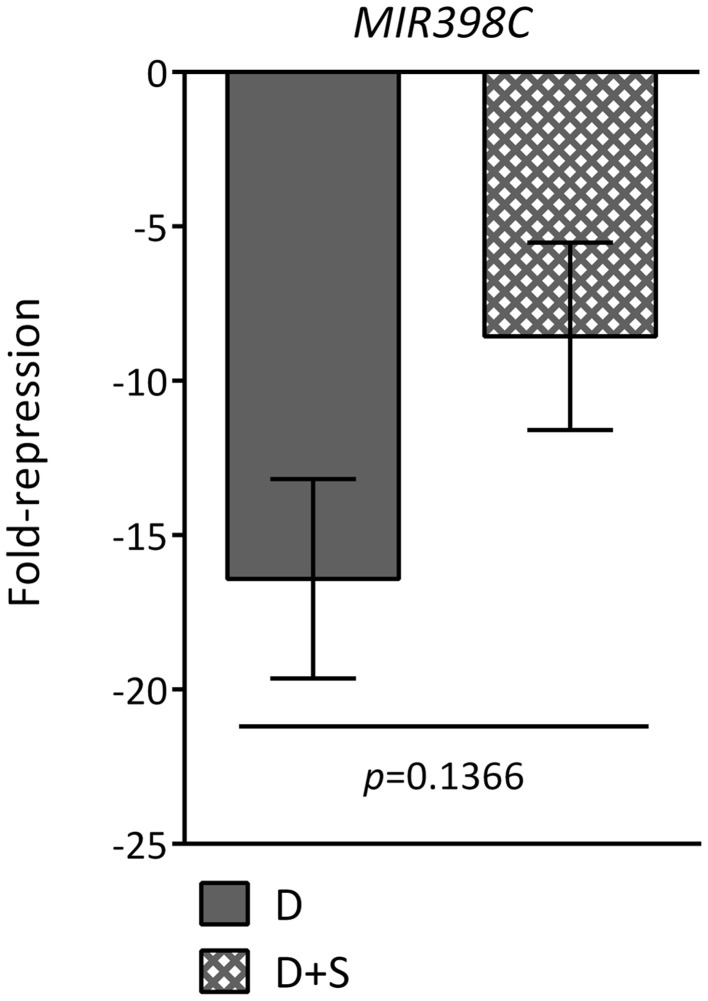
***MIR398C* expression is not significantly affected by glucose.** Dark treatment of leaves results in *MIR398C* repression, but this repression is not significantly altered by the addition of glucose. Values represent fold-repression in the dark (D) and dark+sugar (D+S) relative to the light control. Relative mRNA levels were assessed by qRT-PCR, error bars represent the standard error of the mean (SEM) from at least three independent experiments. *p*-values, paired *t*-test.

**Figure A2:**
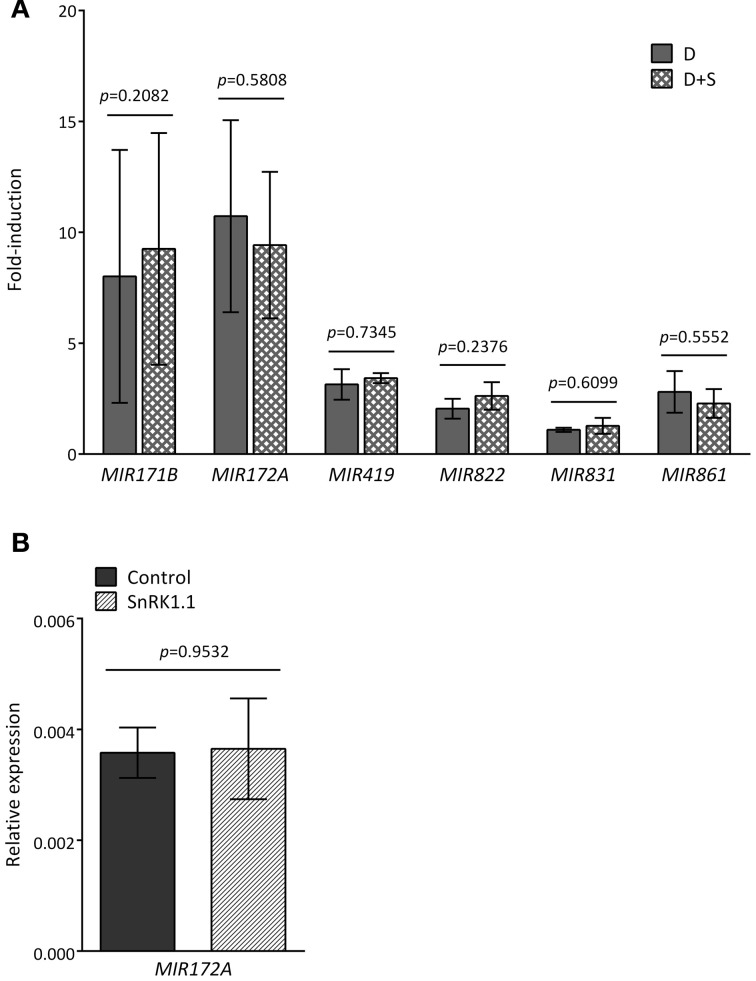
***MIR* gene induction in response to darkness is independent of the energy status and SnRK1 activity. (A)** Increase of *MIR* transcript levels in dark-treated leaves is independent of the energy status. Values represent fold-induction in the dark (D) and dark+sugar (D+S) relative to the light control. **(B)** SnRK1.1 activation in mesophyll protoplasts does not significantly alter *MIR172A* transcript levels. Values represent relative transcript levels upon transient overexpression of SnRK1.1 or control DNA. Relative mRNA levels were assessed by qRT-PCR, error bars represent the standard error of the mean (SEM) from at least three independent experiments. *p*-values, paired *t*-test.

Since the ATH1 chips previously employed in the starvation and SnRK1 transcriptional profiling (Baena-Gonzalez et al., 2007) did not contain probes for the *MIR* genes, and in order to distinguish between an effect caused directly by darkness or indirectly by energy deficit, we tested by quantitative real-time RT-PCR (qRT-PCR) whether the addition of sugar (50 mM glucose) was able to alleviate the effect of the dark stress (Figure 2A). For most of the dark-repressed *MIR* transcripts, namely *MIR157C*, *MIR159A, MIR159B*, *MIR161*, *MIR775A*, *MIR824A*, and *MIR849A*, the presence of sugar could indeed alleviate the dark repression to different extents, suggesting that their expression is partly controlled by the energy status. *MIR398C* was strongly reduced in darkness but this reduction was not significantly affected by glucose (Figure A1). *MIR414* expression was not further explored due to the uncertainties regarding it being a true miRNA (Xie et al., 2005; Rajagopalan et al., 2006). However, for all the induced *MIR* transcripts tested the addition of sugar could not alter the effect of darkness, suggesting that their expression is regulated solely by the absence or presence of light (Figure A2A). As already suggested by the low hybridization signals obtained in the array, the transcript levels of *MIR169E* and *MIR319C* were so low that no reliable quantification by qRT-PCR could be obtained (not shown).

To test whether SnRK1 could cause a reduction in *MIR* transcript levels similarly to dark-induced energy depletion, we induced SnRK1 signaling by transiently overexpressing SnRK1.1 in *Arabidopsis* protoplasts, and measured *MIR* transcript changes by qRT-PCR. Indeed, SnRK1 activation resulted in a clear decrease in *MIR159B, MIR161, MIR775A*, and *MIR824A* (Figure 2B). For *MIR159A*, the results were inconclusive, whereas for *MIR849A* and *MIR157C* the low transcript levels in isolated protoplasts precluded amplification.

SnRK1 activation had no clear effect on *MIR172A* (Figure A2B) or any of the other *MIR* transcripts induced upon exposure to darkness, further supporting that the described induction of *MIR* genes relies on an energy- and SnRK1-independent signaling pathway, whereas the repression of specific *MIR* genes (Figure 2) is partly dependent on the energy status.

### Several mechanisms underlie the dark-induced changes in *MIR* transcripts

**Figure 3:**
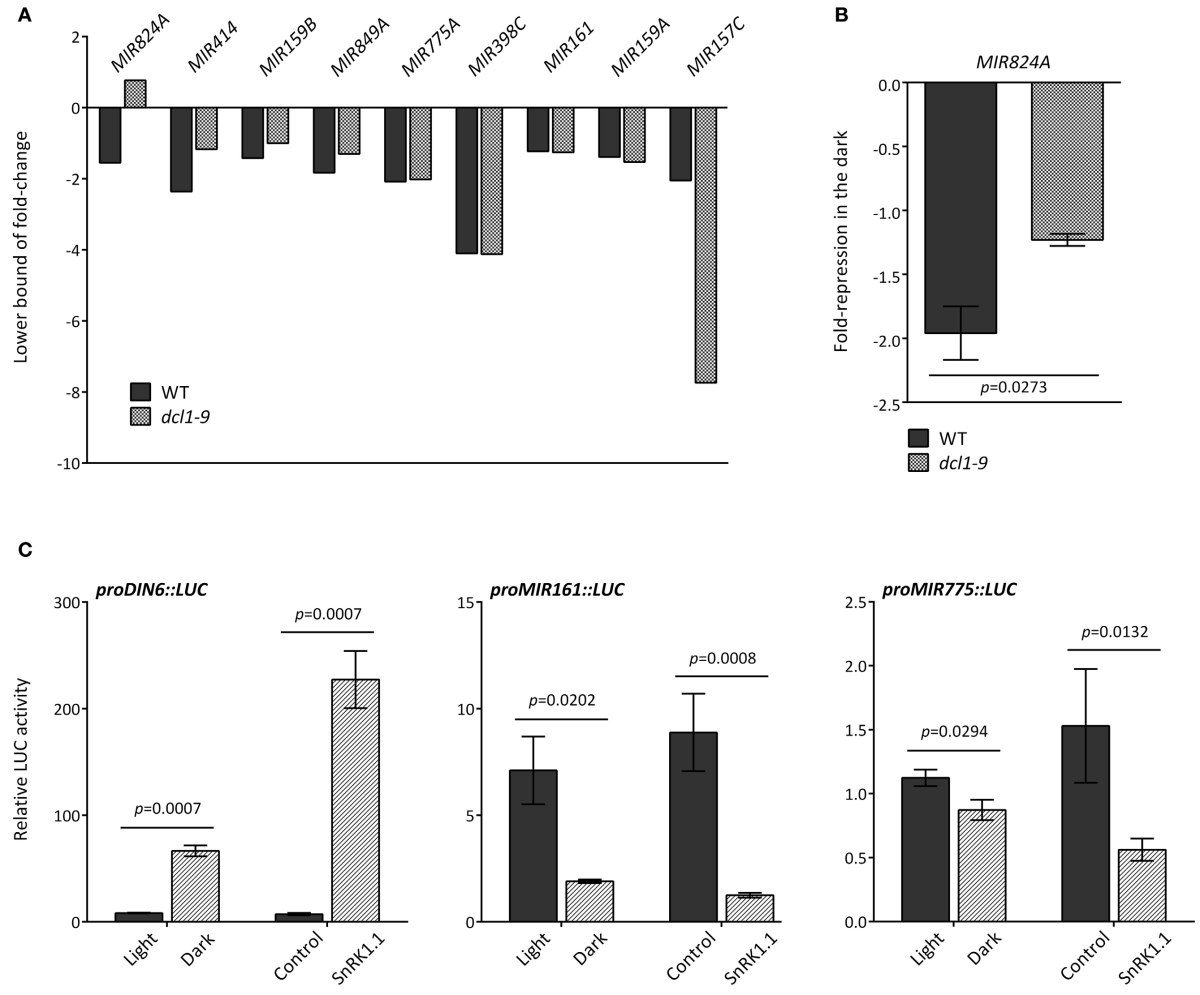
**Different mechanisms underlie the dark-triggered decline of *MIR* transcripts. (A)** Misregulation of specific *MIR* genes in the *dcl1–9* mutant. Fold-reduction of *MIR* transcripts in response to darkness in WT and *dcl1–9* leaves corresponds to the 90% confidence lower bound of fold-change, as calculated with dChip from the microarray hybridization data obtained from detached leaves incubated in the light or in darkness. **(B)** The dark-triggered reduction in *MIR824A* was confirmed by qRT-PCR. Values denote the fold-repression of *MIR824A* in the dark relative to the light in WT and *dcl1–9* leaves. **(C)** The activity of *MIR161* and *MIR775A* putative promoters is reduced by darkness and SnRK1.1 overexpression. LUC activity was measured as readout of promoter activity using the indicated *proMIR::LUC* fusion constructs. Activation of *proDIN6::LUC* is a positive control for activation of the SnRK1 pathway. LUC activities were normalized to GUS activities generated by the co-transfected *UBQ10::GUS* construct that served as an internal transfection control. Error bars represent the standard error of the mean (SEM) from at least three independent experiments. *p*-values, unpaired *t*-test **(B)** or ratio *t*-test **(C)**.

**Figure A3:**
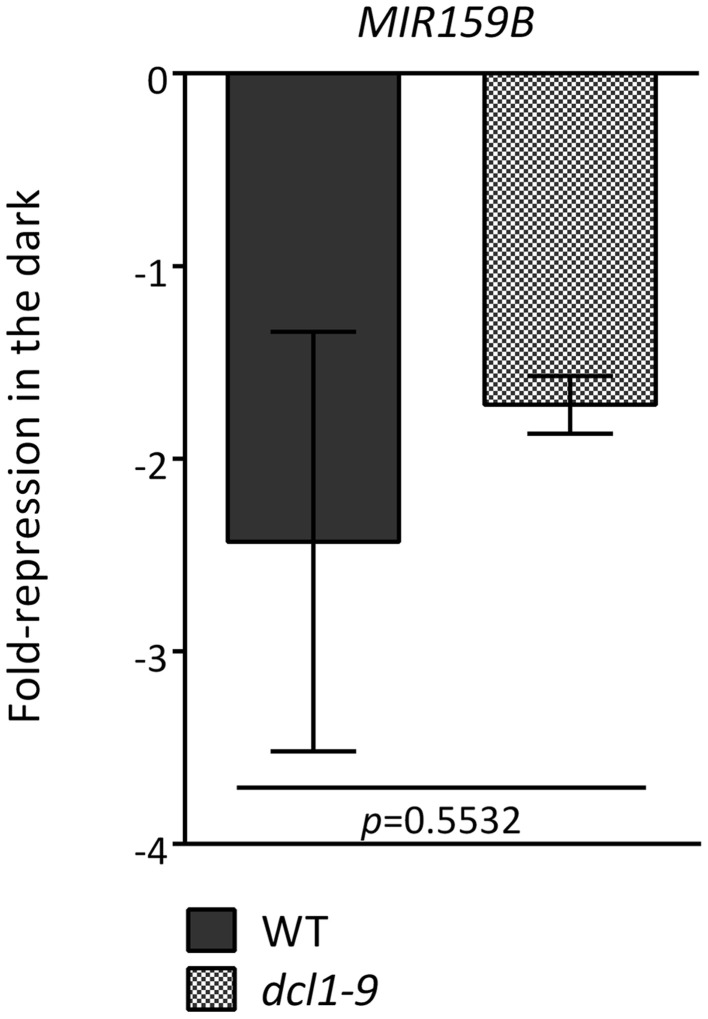
***MIR159B* repression by darkness is not DCL1-dependent.** The dark-triggered reduction in *MIR159B* was measured by qRT-PCR from an independent set of three experiments. Values denote the fold-repression of *MIR159B* in the dark relative to the light in WT and *dcl1–9* leaves. Relative mRNA levels were assessed by qRT-PCR, error bars represent the standard error of the mean (SEM) from at least three independent experiments. *p*-values, unpaired *t*-test

A reduction in *MIR* transcripts could be due to several factors, including increased processing, differential transcript stability and/or decreased promoter activity. For several *MIR* transcripts an altered regulation in *dcl1–9* (greater or equal than 15%) in darkness was indeed detected in the microarray (Figure 3A, Table S3), and could be confirmed by qRT-PCR for *MIR824A* (Figure 3B), but not for *MIR159B* (Figure A3).

**Figure A4:**
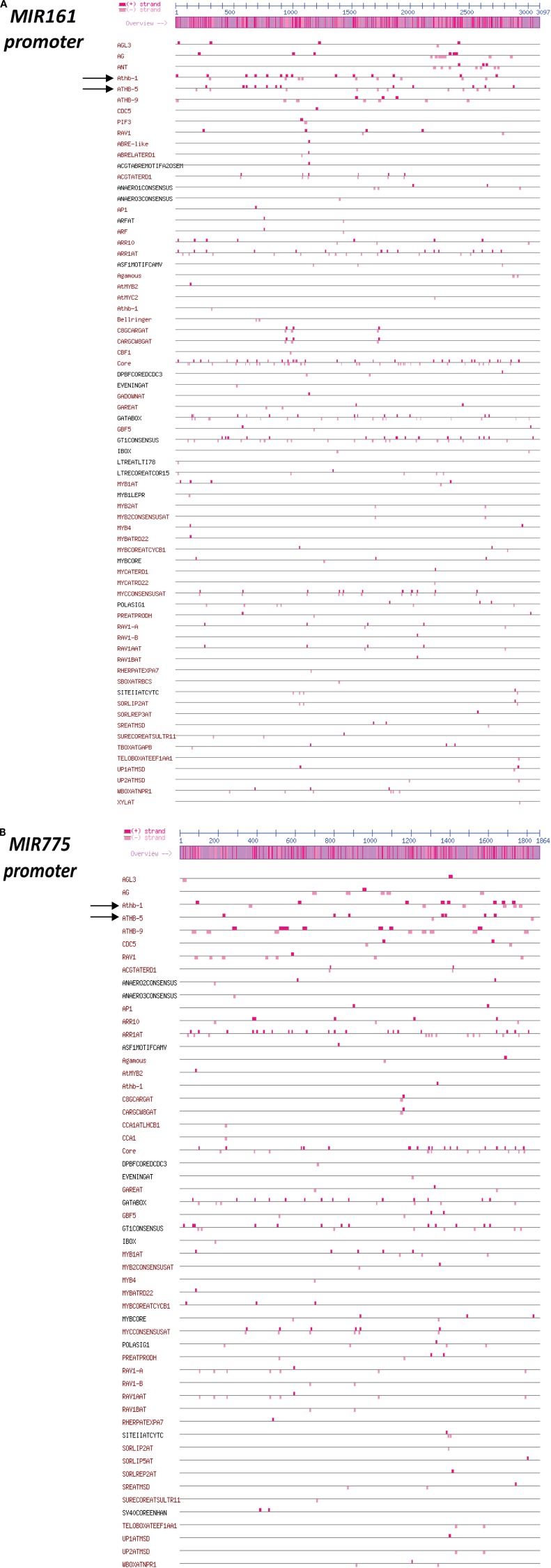
**Promoter analysis of *MIR* genes.** PlantPAN (Plant Promoter Analysis Navigator) *cis*-motif analyses reveal abundant ATHB1 and ATHB5 (indicated with arrows) binding sites in the *MIR161* **(A)** and to a lesser extent in the *MIR775*A **(B)** promoters.

To investigate DCL1-independent differences in *MIR* levels we examined the promoter activity associated with *MIR* transcripts that were similarly reduced by darkness in WT and *dcl1–9* plants. To this end, we cloned the genomic sequences upstream of the predicted fold-back structures of *MIR161* (–3086 bp) and *MIR775* (–1853 bp) and fused it to the *LUC* coding region to generate *MIR promoter*::*LUC* reporters. In transfected mesophyll protoplasts, the SnRK1 reporter *proDIN6::LUC* is activated by darkness and SnRK1.1 overexpression, and serves as a positive control for SnRK1 activation (Figure 3C; Baena-Gonzalez et al., 2007). SnRK1 activation through dark treatment or through SnRK1.1 overexpression resulted in significant repression of both *MIR161* and *MIR775* promoters (Figure 3C), suggesting the regulation of promoter activity by energy deficiency. A preliminary search for *cis*-elements in the promoters of these *MIR* genes using the web-based Plant Promoter Analysis Navigator (PlantPAN) tool (Chang et al., 2008), revealed a high density of two known variants of the homeobox domain leucine zipper class I (HDZipI) promoter motif, ATHB-5 and ATHB1 in the more proximal part of the *MIR161* promoter (1500 bp) (Figure A4A), and to a lesser extent in the *MIR775* promoter (Figure A4B). This is consistent with a report on the enrichment of these motifs in the upstream genomic regions of hypoxia-responsive miRNAs (Moldovan et al., 2010).

### Identification of starvation genes potentially regulated by miRNAs

**Figure 4:**
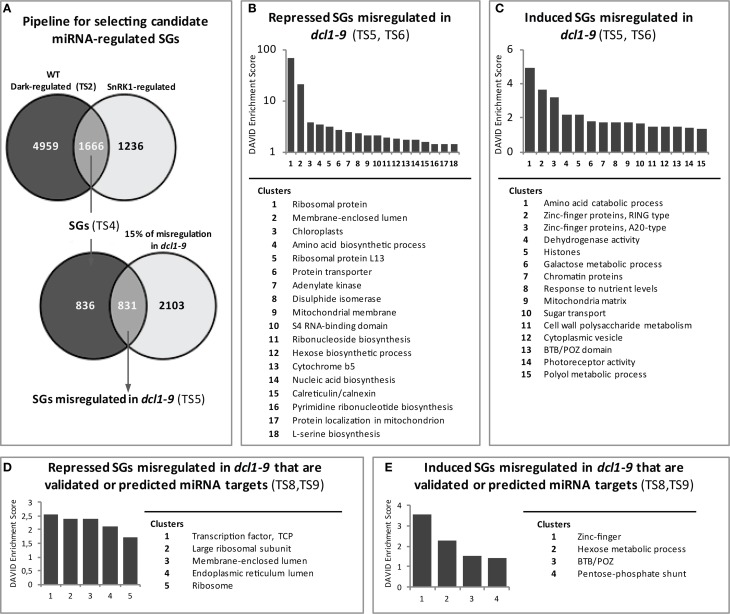
**Identification and functional analysis of potential miRNA-regulated starvation genes. (A)** Pipeline for the identification of starvation genes (SGs) misregulated in the *dcl1–9* mutant. The list of genes with significant differential expression in response to darkness in the WT (“Dark-regulated”) was intersected with that of genes regulated by SnRK1 (Baena-Gonzalez et al., 2007). The response of the overlapping genes (SGs) was examined in the *dcl1–9* mutant and those exhibiting at least 15% misregulation in *dcl1–9* were selected as SGs misregulated in *dcl1–9*. **(B,C)** Functional clustering analysis of SGs misregulated in *dcl1–9* reveals 18 and 15 enriched clusters (enrichment score ≥ 1.3) for the repressed **(B)** and induced **(C)** genes, respectively. **(D,E)** Functional clustering analysis of SGs misregulated in *dcl1–9* that are validated or predicted miRNA targets reveals 5 and 4 enriched clusters (enrichment score ≥ 1.3) for the repressed **(B)** and induced **(C)** genes, respectively. *TS*, Supplementary Table.

To investigate in more detail the possible involvement of miRNAs in the starvation-triggered changes in gene expression, we first generated a list of genes similarly regulated by darkness in the WT (Table S2) and SnRK1 activation (1.5-fold-change; Baena-Gonzalez et al., 2007), which we called “starvation genes” (SGs, Table S4). In Figure 4A a pipeline of the microarray analyses is depicted. The cut-off used in previous studies (2-fold; Baena-Gonzalez et al., 2007) was lowered because of the mild impact expected by transient miRNA regulation, as described in the previous section. We then searched for SGs with partially compromised dark regulation in the *dcl1–9* mutant, using a misregulation cut-off of 15%. Deficient miRNA processing and reduced mature miRNA levels in *dcl1–9* should result in deficient regulation of miRNA targets in this background. Again, the cut-off was selected based on the partial deficiency reported for the *dcl1–9* mutant in response e.g., to *flg22* peptide treatment or abiotic stress treatments (Navarro et al., 2006; Laubinger et al., 2010) (Figure 1). Furthermore, *dcl1–9* is not a null, but a hypomorphic mutant, and hence miRNA accumulation in this background is not fully abolished (Vazquez et al., 2008). Of the 1666 SGs (Table S4), 831 genes had a compromised response in the *dcl1–9* mutant (Table S5), suggesting that their response to starvation could be mediated by miRNAs. We call these “SGs misregulated in *dcl1–9*.” To gain a global functional view of these genes we assigned GO terms and performed a functional clustering analysis using DAVID (Huang Da et al., 2009). The functional categories could be resolved into 15 and 18 enriched clusters for the upregulated and downregulated genes, respectively, with enrichment scores equal or higher than 1.3 (Huang Da et al., 2009) (Figures 4B,C, Table S6). The enrichment score for several of the repressed clusters was remarkably high, with cluster 1 and cluster 2 having 67.1 and 20.6, respectively, whereas the enrichment score for the highest ranking cluster of upregulated genes was 4.9. For the repressed SGs misregulated in *dcl1–9*, the main clusters relate to ribosomal proteins and translation, organelle function (mitochondria, ER, and plastid), protein transport and folding, redox signaling, and nucleic acid metabolism, whereas for the induced SGs misregulated in *dcl1–9*, the main clusters relate to amino acid catabolic processes, protein degradation, chromatin remodeling, sugar and fatty acid metabolism, and autophagy. Interestingly, a large number of SGs misregulated in *dcl1–9* exhibit altered expression in the *ago1–9* mutant (Ronemus et al., 2006), supporting the hypothesis that they are under miRNA control (Table S5). The effect is clearly more pronounced for the repressed SGs, out of which 12.6% are upregulated more than 1.5-fold in *ago1–9*. For the induced SGs only 4.6% are downregulated more than 1.5-fold in *ago1–9* (Table S5). This differential impact is not due to a differential coverage of these genes in the Arabidopsis 8K chip employed in the Ronemus study, since that platform covers 38 and 43% of the induced and repressed SGs misregulated in *dcl1–9*, respectively. Importantly, the effect of the *ago1–9* mutation is clearly more visible in the 9d- than in the 21d-old seedling samples (Table S5). Given the nature of the affected genes, mostly involved in translation, organelle function and metabolism, one could argue that a deficiency in their regulation may be more apparent in seedlings with higher metabolic activity and growth rates than in mature leaves. Similarly, the age of the plants could also explain why only a few of these genes exhibited a significantly altered expression under control basal conditions in our or Ronemus *dcl1–9* samples (35d-, and 21d-old plants, respectively; Table S5). On the other hand, some of the SGs that were not misregulated in the *dcl1–9* mutant had also altered expression levels in the 9d-old *ago1–9* samples (Table S4), again suggesting an age effect, a weak effect of the *dcl1–9* mutation on the corresponding miRNAs as compared to *ago1–9* (Ronemus et al., 2006), a dependency on DCL4 or simply the involvement of small RNAs other than miRNAs.

**Figure A5:**
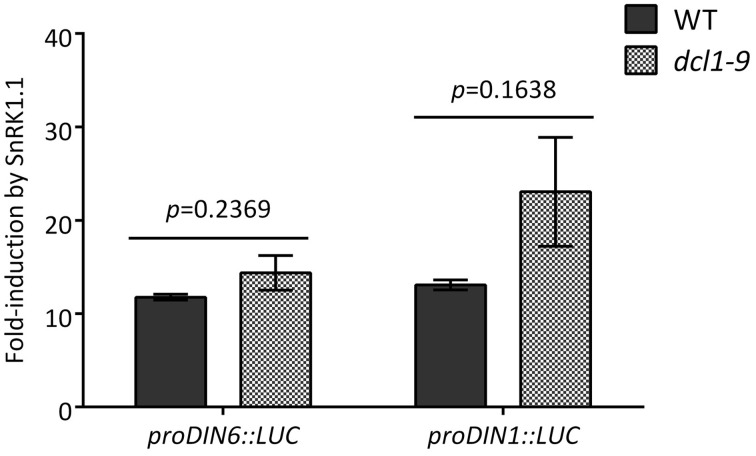
**SnRK1 activation is not impaired in the *dcl1–9* mutant.** SnRK1 activation through transient overexpression of SnRK1.1 was comparable in protoplasts of WT and *dcl1–9* plants. The activation of the *proDIN6::LUC* and *proDIN1::LUC* reporters was used as readout of SnRK1 activity. LUC activities were normalized to GUS activities generated by the co-transfected *UBQ10::GUS* construct that served as an internal transfection control. Values represent the fold-induction of GUS-normalized LUC activities in response to SnRK1.1 overexpression relative to transfection with control DNA. Error bars represent the standard error of the mean (SEM) from at least three independent experiments. *p*-values, paired *t*-test.

Having established the dependency of SG regulation on DCL1, we first asked whether deficient SG regulation in the *dcl1–9* mutant was the consequence of a general deficiency in SnRK1 signaling. Despite being clearly induced by darkness many of the established SnRK1 target genes (Baena-Gonzalez et al., 2007), like *DIN6* (At3g47340) and *DIN1* (At4g35770), accumulated to lower levels in the *dcl1–9* mutant (Table S5). To test whether the cause for this was a deficient SnRK1 activation in the *dcl1–9* mutant, we measured *LUC* induction from the *proDIN6::LUC* and *proDIN1::LUC* reporters as readout of SnRK1 activity (Baena-Gonzalez et al., 2007; Ramon et al., in press) in transfected mesophyll cells from WT and *dcl1–9* leaves. Reporter genes were induced to a similar extent in WT and *dcl1–9* cells, indicating no major differences in SnRK1 activation between the two genotypes (Figure A5), and suggesting other mechanisms, such as transcript stability, behind differential transcript accumulation.

As a means to discern between direct and secondary miRNA targets, we next asked whether the identified SGs misregulated in *dcl1–9* were validated or predicted miRNA targets. To this end we employed a recently published list (Folkes et al., 2012) of validated and predicted targets compiled from literature and from public databases [ASRP and *Arabidopsis* MPSS and PARE databases (Nakano et al., 2006; Backman et al., 2008; German et al., 2008)]. In addition, we used the *Arabidopsis* miRNA sequences deposited in the miRBase database (Kozomara and Griffiths-Jones, 2011) and predicted further targets employing the psRNAtarget (Dai and Zhao, 2011), TargetFinder (Allen et al., 2005; Fahlgren et al., 2007), and the UEA Plant Target Prediction tools (Moxon et al., 2008). Altogether, the published list of validated and predicted targets combined with the results of the prediction tools using TAIR10 annotation as a reference yielded a total of 4961 transcripts with proved or potential miRNA regulation (Table S7). The intersection of this list with that of SGs misregulated in *dcl1–9* yielded a total of 155 genes (Table S8). We call these “SGs misregulated in *dcl1–9* that are validated or predicted miRNA targets.”

Functional analyses (Table S8) and clustering (Table S9) of the SGs misregulated in *dcl1–9* with validated or potential miRNA regulation revealed, in addition to the already observed “ribosomal proteins and translation” and “ER and mitochondrial function” clusters, the enrichment of the TCP (Teosinte branched1, Cycloidea, and Proliferating cell nuclear antigen) motif (Table S9, Figures 4D,E), present in TCP2 (At4g18390), TCP4 (At3g15030), and a third TCP-family member (At1g35560) of unknown function. Due to the limited size of the gene set, many of the genes remain unclustered. This is the case for numerous genes encoding stress-related and signaling components with known or potential connection to the SnRK1 pathway (Table S8). The induced genes include for example *SnRK2.10* (At1g60940), *ATG8E* (At2g45170), and *AKINβ1* (At5g21170), involved in salt stress, autophagy and SnRK1 signaling, respectively, (Polge and Thomas, 2007; Li and Vierstra, 2012; McLoughlin et al., 2012). The unclustered repressed genes, on the other hand, include for example *SnRK3.10* (At3g23000), *MYB75* (At1g56650), and *Hsp70-15* (At1g79920), involved in Ca^2+^-signaling and stress tolerance, anthocyanin synthesis, and the heat response (Teng et al., 2005; Weinl and Kudla, 2009; Jungkunz et al., 2011).

### Target mRNAs are repressed by miRNAs in response to energy depletion and SnRK1 activation

In order to validate some of the predicted regulatory interactions, we selected specific target genes to experimentally confirm their energy-, SnRK1-, and DCL1/miRNA-dependent regulation in response to darkness. To this end we chose *TCP2* and *TCP4* as representative enriched components with validated miRNA regulation (miR319; Palatnik et al., 2003) and with a misregulation of over 30% in the *dcl1–9* mutant (Table S8). Although we were not able to amplify the *MIR319* transcript by qRT-PCR, the microarray experiment showed a moderate increase for this precursor (Table S3), and the induction of both *MIR319* and mature miR319 in response to multiple types of stress has also been reported in *Arabidopsis*, rice and sugar cane (Sunkar and Zhu, 2004; Liu et al., 2008; Lv et al., 2010; Zhang et al., 2011; Thiebaut et al., 2012; Zhou et al., 2013). We also selected *Hsp70-15*, predicted to be targeted by miR831 and with a much less severe misregulation in *dcl1–9* (19%, Table S8) as a way to validate our approach and cut-off.

**Figure 5:**
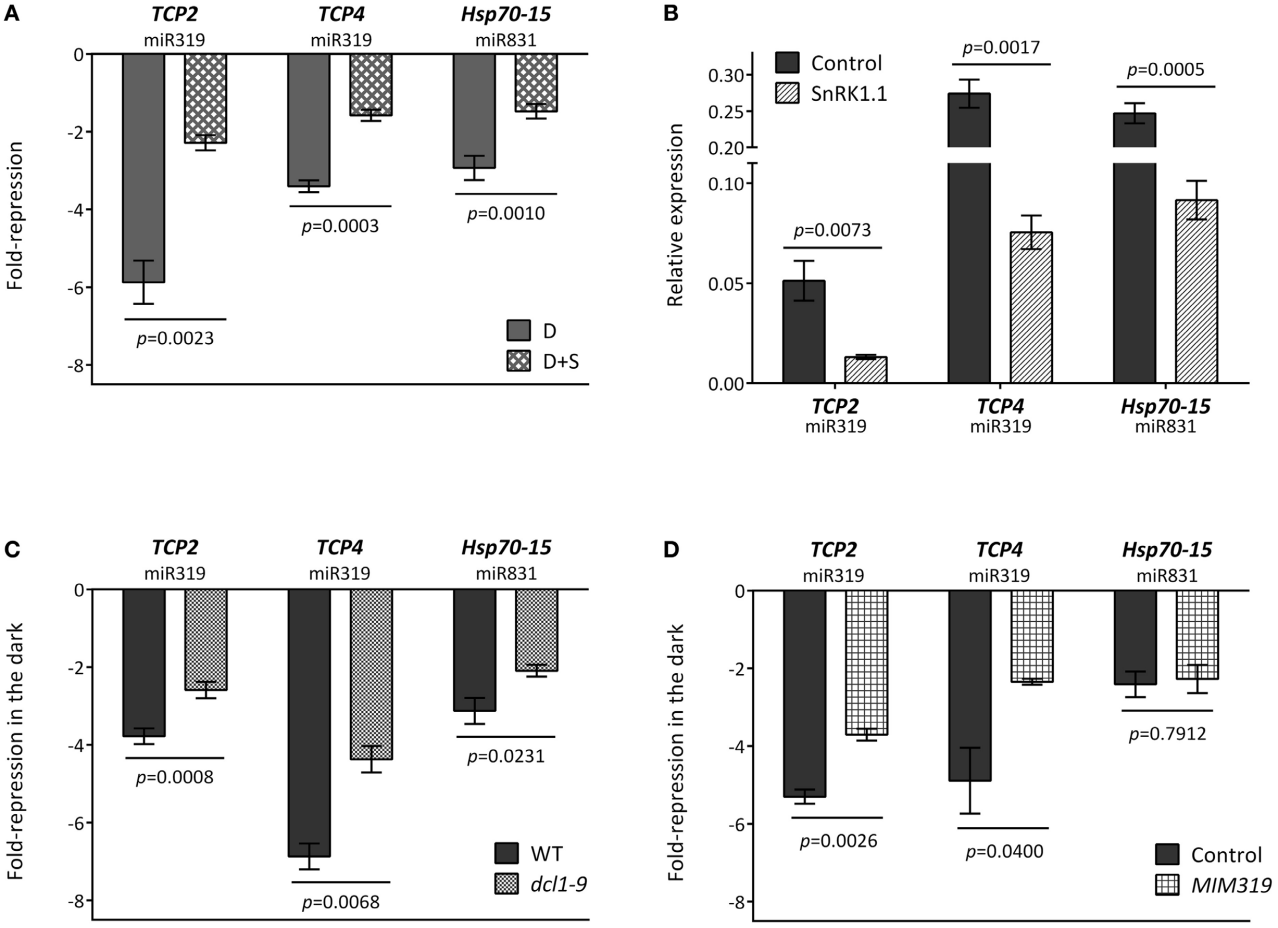
**Repression of *TCPs* by energy deprivation requires miRNA function. (A)** Repression of *TCPs* and *Hsp70-15* in dark-treated leaves is dependent on the energy status. Values represent fold-repression in the dark (D) and dark+sugar (D+S) relative to the light control. **(B)** SnRK1.1 overexpression in mesophyll protoplasts causes a reduction in *TCP* and *Hsp70-15* levels. Values represent relative transcript levels upon transient overexpression of SnRK1.1 or control DNA. **(C)** Repression of *TCPs* and *Hsp70-15* by energy deprivation is partly compromised in the *dcl1–9* mutant. **(D)** Repression of *TCPs* but not of *Hsp70-15* by energy deprivation is partly compromised in *MIM319* plants. Values in **(C)** and **(D)** denote the fold-repression of transcripts in dark-treated as compared to light-treated leaves in the indicated genotypes. Relative mRNA levels were assessed by qRT-PCR, error bars represent the standard error of the mean (SEM) from at least three independent experiments. *p*-values, paired **(A,B)** or unpaired *t*-test **(C,D)**.

**Figure A6:**
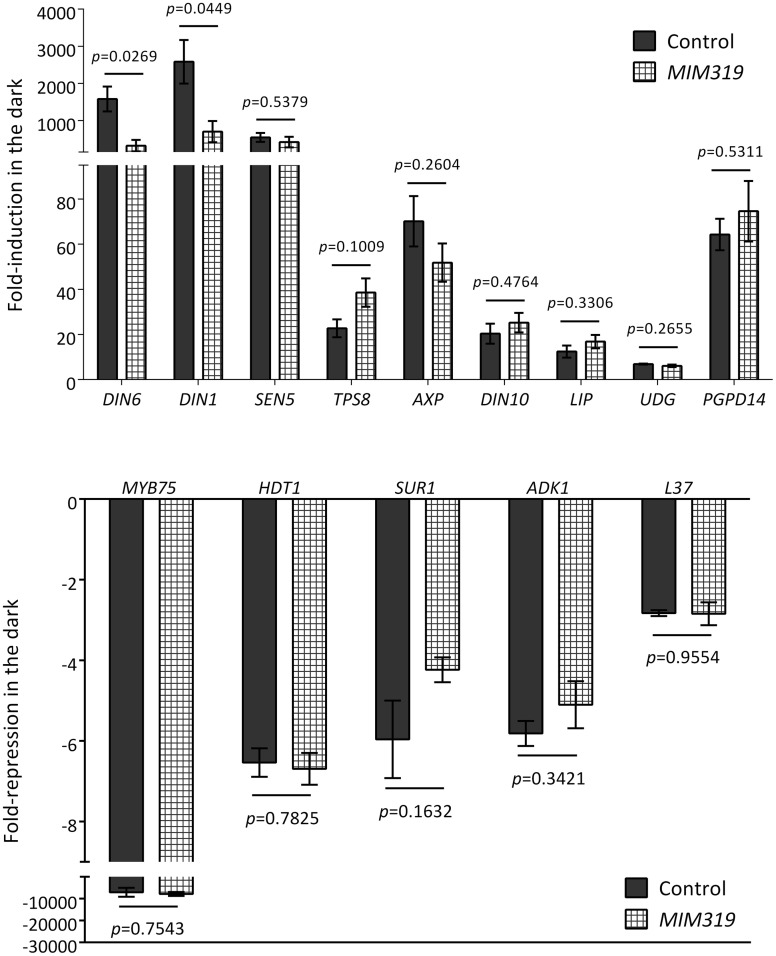
**SnRK1 signaling is not generally compromised in *MIM319* plants.** Dark-treated leaves of *MIM319* plants show a normal regulation of most SnRK1 marker genes (Baena-Gonzalez et al., 2007), with the exception of *DIN6* and *DIN1*, whose induction is compromised. Values denote the fold-change of transcripts in the dark as compared to light in control plants expressing the empty vector and in *MIM319* plants. Relative mRNA levels were assessed by qRT-PCR, error bars represent the standard error of the mean (SEM) from at least three independent experiments. *p*-values, unpaired *t*-test.

As shown in Figure 5A, incubation in darkness triggered a clear reduction in the three selected targets, and this reduction was partly relieved in the presence of sugar, suggesting that the energy status played a role in the changes observed in darkness. As expected from this observation, these transcripts were also repressed by SnRK1 activation (Figure 5B). Most importantly, the dark- and SnRK1-induced repression of these mRNAs was partially compromised in the *dcl1–9* mutant (Figure 5C), in agreement with the hypothesis that the dark-repression of these mRNAs requires miRNA function. To confirm the dependency of the dark repression on miRNAs and to rule out other potential miRNA-independent DCL1 functions, we analyzed the dark repression of *TCP4* and *TCP2* in plants overexpressing a miR319 target mimic and hence displaying reduced miR319 activity (*MIM319*; Franco-Zorrilla et al., 2007). The repression of both *TCP2* and *TCP4*, but not that of the putative miR831 target *Hsp70-15* was clearly impaired in *MIM319* plants, indicating that miR319 is required for the repression of *TCP*s in response to energy deprivation (Figure 5D). Importantly, this was not due to a general impact on SnRK1 activity, since a large number of induced and repressed SnRK1 marker genes [Table S2 in Baena-Gonzalez et al. (2007)] either induced or repressed by starvation and SnRK1 displayed a normal response in the *MIM319* plants (Figure A6). Unexpectedly, and similarly to what was observed in the *dcl1–9* mutant, *DIN6* and *DIN1* accumulated to lower levels in *MIM319* plants, suggesting a possible involvement of miR319 or its TCP targets in *DIN* gene regulation.

## Discussion

**Figure 6:**
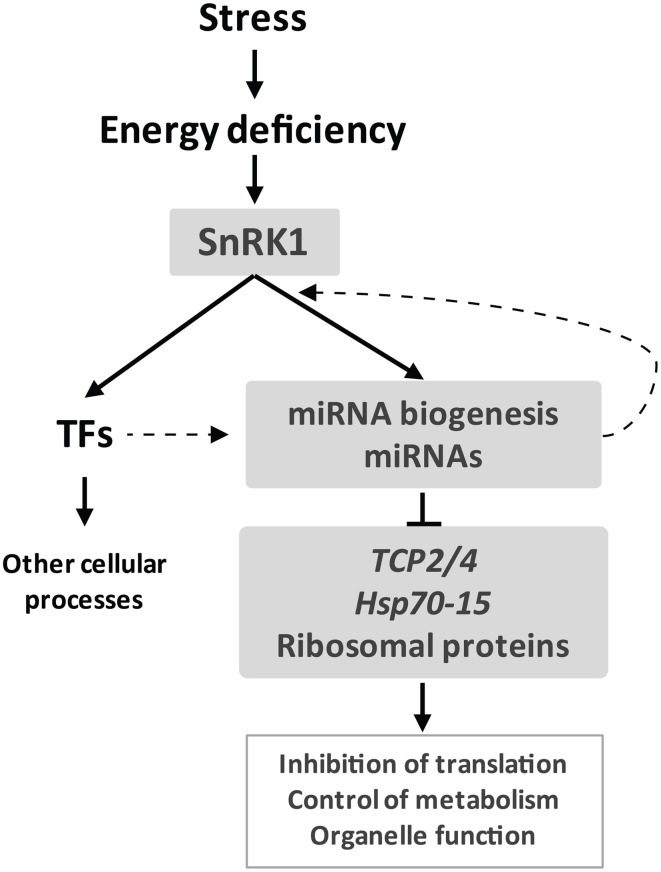
**miRNAs are a novel component of the starvation response.** Stress-derived energy deficiency activates the SnRK1 protein kinase which leads to a major transcriptional reprogramming partly *via* transcription factors and partly *via* miRNAs. SnRK1 activity may impact miRNA function at several levels, including regulation of *MIR* promoter activity. Components of the miRNA pathway may also influence the SnRK1 transcriptome e.g., through changes in transcript stability. miRNAs contribute to SnRK1 signaling mainly through repression of gene expression, targeting *TCP*s and *Hsp70-15*, and possibly impacting major cellular processes like translation.

A growing number of studies implicate plant miRNAs in the response to nutrients as well as abiotic and biotic stress (Ruiz-Ferrer and Voinnet, 2009; Sunkar et al., 2012). SnRK1 protein kinases, on the other hand, play a major role in the survival of plants to adverse conditions through the extensive regulation of metabolism and transcription in response to stress-derived energy deprivation. Comparison of the transcriptional response of WT and *dcl1–9* plants to unpredicted darkness uncovered a partial deficiency in the starvation response of *dcl1–9*, which may translate in a diminished ability of the mutant to withstand stress conditions, similarly to plants with altered levels of specific miRNAs or with a general deficiency in miRNA biogenesis (Sunkar et al., 2012; Zhan et al., 2012). One has to bear in mind though that not all miRNAs are processed by DCL1, and for example miR822 and miR839, processed by DCL4, accumulate to WT levels in the *dcl1–9* mutant (Rajagopalan et al., 2006). Nevertheless, given the major role of DCL1 in miRNA biogenesis the deficient starvation response of *dcl1–9* may suggest that miRNAs contribute to the gene expression reprogramming triggered by SnRK1 kinases (Figure 6). The reduced ability of *MIM319* plants to repress *TCP*s demonstrates this in the case of *TCP* regulation by miR319 (Figure 5D). Even though the *dcl1–9* mutation does not impair SnRK1 activation (Figure A5), many of the SnRK1 target genes show deficient accumulation in response to darkness in the mutant, suggesting that DCL1 contributes directly or indirectly to the termination or stability of these transcripts. Intriguingly, DCL4 was recently implicated in transcriptional termination in Arabidopsis (Liu et al., 2012) and the Microprocessor complex was reported to impact transcript stability in a miRNA-independent manner in mammals (Han et al., 2009; Knuckles et al., 2012). Transcript stability, on the other hand, has been shown to play a role in the shaping of the *Arabidopsis* transcriptome in response to cold stress (Chiba et al., 2013).

Our results suggest several possible layers of regulation of miRNA action in response to energy deficiency. On one hand the *MIR* transcripts of several miRNAs respond to varying degrees to the energy status and SnRK1 activity (Figure 2). Even though these are not the active molecules, changes in *MIR* expression may reflect the engagement of the corresponding mature miRNAs in gene regulation. In some cases, miRNA accumulation may be accompanied by an increase in the levels of the corresponding *MIR* transcript as a result of a strong transcriptional activation. In other cases, enhanced accumulation of mature miRNAs may be accompanied by a decrease in the corresponding *MIR* transcripts presumably as a result of enhanced processing or as a result of negative feedback. For most of the *MIR* transcripts that accumulate differentially in response to darkness (Figure 2), the corresponding mature miRNA was reported to be significantly induced by inhibition of mitochondrial electron transport and by hypoxia (Table S3) (Moldovan et al., 2010), another stress condition known to activate SnRK1 (Baena-Gonzalez et al., 2007). Nevertheless, miRNA accumulation is not always correlated with *MIR* levels, indicating that other mechanisms operate to regulate miRNA activity under those conditions, and factors like miRNA stability, target levels and feedback loops may be invoked to explain the observed discrepancies (Schwab et al., 2005; Franco-Zorrilla et al., 2007; Reyes and Chua, 2007; Ramachandran and Chen, 2008; Laubinger et al., 2010).

Different mechanisms of regulation of miRNA activity may operate in response to different signals and depending on the miRNA, tissue, and developmental stage. The importance of transcriptional regulation has been shown e.g., for miR398 and miR408, whose *MIR* transcripts are induced in response to copper deficiency and sucrose through the SPL7 transcription factor, and this induction is abrogated in plants depleted of SPL7 (Dugas and Bartel, 2008; Yamasaki et al., 2009; Ren and Tang, 2012). The fact that glucose did not have a significant effect on the strong dark repression of *MIR398C* may be due to different kinetics of repression/activation as compared to other dark-repressed *MIR*s or may suggest the involvement of a sucrose-specific signaling pathway (Rolland et al., 2006).

An effect on promoter activity was observed for *MIR161* and *MIR775*, both repressed by darkness and SnRK1 activity (Figure 3C). Considering that the corresponding mature miRNAs accumulate in response to hypoxia (Moldovan et al., 2010), the decrease in promoter activity is more likely to reflect a negative feedback loop coupled to miRNA activity or more generally to the starvation response. Given that control of promoter activity by miRNAs would imply an altered *MIR* transcript accumulation in the *dcl1–9* mutant, and that *MIR161* and *MIR775* are reduced to a similar extent in *dcl1–9*, we favor the second hypothesis. In this context, Moldovan et al. (2010) performed an *in silico* search for enriched *cis*-elements in the promoters of *MIR* genes with ≥1.5 fold-changes in response to hypoxia and found that two known variants of the homeobox domain leucine zipper class I (HDZipI) promoter motif, ATHB-5 and ATHB-1 were significantly overrepresented. Such elements seem indeed to be enriched in the more proximal part of the *MIR161* promoter (1500 bp), although for *MIR775* the same is not clear (Figure A4). Whether these transcription factors are indeed involved in *MIR* gene regulation during stress and whether they are under SnRK1 control remains to be determined.

In addition to promoter activity, other mechanisms for *MIR* regulation are also possible, and both intron retention and alternative splicing have recently been demonstrated to play a role in miRNA biogenesis and function (Yan et al., 2012; Jia and Rock, 2013). Differential processing efficiency could also be due to the action of ancillary proteins, as shown for the *C. elegans* RNA binding protein Lin-28, which blocks specifically the processing of *let-7* pri-miRNA in stem cells (Newman et al., 2008; Piskounova et al., 2008; Rybak et al., 2008; Viswanathan et al., 2008) or for the *Arabidopsis* RNA binding protein FCA, which drives differential miRNA processing in response to temperature (Jung et al., 2012).

For *MIR824A* the observed decline in darkness was partially compromised in the *dcl1–9* mutant (Figures 3A,B), which may be suggestive of enhanced *MIR824A* processing by DCL1 under energy starvation conditions. It is also possible that *MIR824A* is subject to negative feedback by its own miRNA, as has been proposed (German et al., 2008; Meng et al., 2010). In this context, several of the affected *MIRs*, including *MIR824A*, are predicted to be targeted by their own miRNA (Table S3), but whether self-regulation plays indeed a role in their expression awaits further investigation. Alternatively, these differences may be explained by other potential miRNA-independent DCL1 functions on transcript termination or stability, as already discussed.

Regardless of the mode of regulation, our study has uncovered a set of 831 SGs misregulated in the *dcl1–9* mutant (Table S5). Of these 12.6% of the repressed and 4.6% of the induced genes were up- and downregulated, respectively, in independent experiments in the *ago1–9* mutant (Ronemus et al., 2006), reinforcing the hypothesis of these genes being under miRNA control. However, from the total set of SGs misregulated in *dcl1–9*, only 19%, including *TCP2* and *TCP4*, are validated or predicted miRNA targets (Table S8). Even though it is possible that the prediction power of the used tools is not sufficient to identify all targets, we favor the hypothesis that a fraction of the SGs require miRNA action but are not themselves under direct miRNA control. On the other hand, despite the clear energy-dependency of *MIR159B*, *MIR161*, *MIR824A*, and *MIR775*, only two predicted targets for miR159ab (At4g15530 and At2g41600) are amongst the SGs misregulated in *dcl1–9*. This may be partly due to the stringent multilayered filtering applied or to the general performance of microarrays analyses, which may be limited for detecting differential expression at low expression levels (Wang et al., 2006). Alternatively, this could be due to the weak nature of the *dcl1–9* mutant, to regulation of miRNA activity downstream of DCL1 (Earley et al., 2010; Earley and Poethig, 2011; Wang et al., 2011; Alonso-Peral et al., 2012; Brodersen et al., 2012), or to the engagement of these miRNAs in translational attenuation (Brodersen et al., 2008; Lanet et al., 2009; Yang et al., 2012).

The functional clusters of SGs most significantly affected by the miRNA pathway correspond to repressed genes related to ribosomal proteins and translation, as well as organelle function (mitochondria, ER, and plastid) (Figure 6). This is in accordance with the role of SnRK1 as a repressor of biosynthetic processes and as a modulator of energy metabolism (Baena-Gonzalez et al., 2007; Baena-Gonzalez and Sheen, 2008), and suggests that miRNAs contribute to SnRK1 signaling mainly through downregulation of gene expression. Interestingly, the *ago1–9* mutation was also reported to cause a major deregulation of ribosomal protein genes (Ronemus et al., 2006), in agreement with the view that translation-related components are under miRNA control. The most prominent cluster of repressed SGs misregulated in *dcl1–9* that are validated or predicted miRNA targets contained the TCP transcription factors. In addition to their role as negative regulators of cell proliferation and growth during leaf development (Palatnik et al., 2003), recent reports have implicated TCPs in the plant response to environmental stress and energy metabolism (Robison et al., 2009; Giraud et al., 2010; Zhou et al., 2013). TCPs were shown to regulate nuclear-encoded mitochondrial genes and contribute in this way to the control of mitochondrial function, in particular TCA cycle function and core energy metabolism/amino acid metabolism (Giraud et al., 2010). Perhaps related to this, and in accordance with our results, TCPs were shown to be downregulated in mutants of the mitochondrial ATP synthase, suggesting that they could be controlled by ATP generation by mitochondria (Robison et al., 2009). Finally, numerous signaling components with known or potential connection to the SnRK1 pathway, such as *AKINβ1*, *SnRK2.10*, or *SnRK3.10*, were also uncovered as candidate miRNA targets. Further studies will be required to validate these predictions and to explore their relevance to energy signaling.

Noteworthy, a link between miRNAs and nutrients and metabolism seems to exist also in animal systems, and miRNAs have been shown to play a role in insulin signaling, glucose homeostasis and nutrient sensing (Bhattacharyya et al., 2006; Poy et al., 2007). More recently, AMPK activation through AICAR treatment was reported to induce the differential accumulation of multiple miRNAs (Liu et al., 2013). miRNAs may therefore represent common elements in diverse organisms for restoring homeostasis following stress. Further work is required to establish more precise mechanistic connections between SnRK1 activity and miRNA function and to characterize the cellular processes repressed by the SnRK1/miRNA axis in order to better understand how SnRK1 activity is translated into enhanced stress tolerance and modified growth and development.

### Conflict of interest statement

The authors declare that the research was conducted in the absence of any commercial or financial relationships that could be construed as a potential conflict of interest.

## Supplementary material

Table S1 **List of primers used in this study**.

Table S2 **Microarray experiment of WT (A) and *dcl1–9* (B) leaves under control or dark conditions**. *Genes significantly changed at least 1.2-fold (lower confidence bound of fold-change)*.

Table S3 ***MIR* genes with significant differential expression in response to darkness**.

Table S4 **List of starvation genes (SGs)**.

Table S5 **List of SGs misregulated in *dcl1–9***.

Table S6 **Gene description, functional annotation and cluster enrichment analyses of SGs misregulated in *dcl1–9***.

Table S7 **List of validated and predicted miRNA targets**.

Table S8 **List of SGs misregulated in *dcl1–9* that are validated or predicted miRNA targets**.

Table S9 **Gene description, functional annotation and cluster enrichment analyses of SGs misregulated in *dcl1–9* that are validated or predicted miRNA targets**.
